# Development and Validation of a Cystatin C-based Staging of AKI in Critically Ill Patients

**DOI:** 10.1016/j.ekir.2026.106514

**Published:** 2026-03-25

**Authors:** Johanna Helmersson-Karlqvist, Liisa Byberg, Johan Ärnlöv, Max Bell, Johan Mårtensson, Alain Dardashti, Maria K. Svensson, Anders Larsson, Michael Marks-Hultström, Miklos Lipcsey

**Affiliations:** 1 Section of Clinical Chemistry, Department of Medical Sciences, Uppsala University, Uppsala, Sweden; 2 Section of Medical Epidemiology, Department of Surgical Sciences, Uppsala University, Sweden; 3 Section of Care Sciences and Society, Department of Neurobiology, Karolinska Institute; 4 Clinical Research Center, Falun, Region Dalarna, Uppsala University; 5 School of Health and Social Studies, Dalarna University, Falun, Sweden; 6 Department of Physiology and Pharmacology, Karolinska Institute, Stockholm, Sweden; 7 Department of Perioperative Medicine and Intensive Care, Karolinska University Hospital, Stockholm, Sweden; 8 Department of Cardiothoracic and Vascular Surgery, Anesthesia and Intensive Care, Skane University Hospital, Lund, Sweden; 9 Section of Renal Medicine, Department of Medical Sciences, Uppsala University, Uppsala, Sweden; 10 Uppsala Clinical Research Center, Uppsala, Sweden; 11 Anesthesiology and Intensive Care Medicine, Department of Surgical Sciences, Uppsala University, Uppsala, Sweden; 12 Section of Integrative Physiology, Department of Medical Cell Biology, Uppsala University, Uppsala, Sweden; 13 Hedenstierna Laboratory, Department of Surgical Sciences, Uppsala University, Uppsala, Sweden

**Keywords:** acute kidney injury, clinical staging, creatinine, cystatin C, glomerular filtration rate

## Abstract

**Introduction:**

Acute kidney injury (AKI) criteria and staging are based on serum creatinine and urine output, but serum cystatin C performs better at estimating glomerular filtration rate (GFR) in critically ill patients. Accordingly, a cystatin C-based AKI staging system was developed and its performance studied in critically ill patients.

**Methods:**

AKI stages according to Kidney Disease: Improving Global Outcomes (KDIGO) creatinine criteria were converted to corresponding cystatin C-based stages using 14-day mortality in 9424 critically ill patients from 3 Swedish hospitals followed for 5.6 years (median interquartile range: 2.8–7.2). Model performance was evaluated using Cox regression on long-term mortality adjusted for age, gender, comorbidities, and unit type. An independent cohort (*n* = 434) was used for validation.

**Results:**

KDIGO stages corresponded to the following: Stage 1: increase in cystatin C 1.40 to 1.59 times baseline within 7 days or ≥ 0.44 mg/l within 48 hours; Stage 2: 1.60 to 2.09 times baseline; and Stage 3: above 2.10 times baseline or ≥ 2.80 mg/l. Cystatin C-based versus creatinine-based staging identified 11% more AKI and 10% more Stage 3. Patients reclassified to AKI by cystatin C from no AKI had a higher risk of death of 1.36 (1.24–1.49), whereas those reclassified vice versa had a lower risk 0.71 (0.56–0.91). These findings were consistent irrespective of infection status for 30-day mortality. In the validation cohort, reclassification to a higher stage by cystatin C was an independent predictor of increased risk of death.

**Conclusion:**

In critically ill patients, cystatin C-based staging identified more AKI than KDIGO criteria, and these patients had increased short- and long-term mortality.

## Graphical abstract

**Figure:**
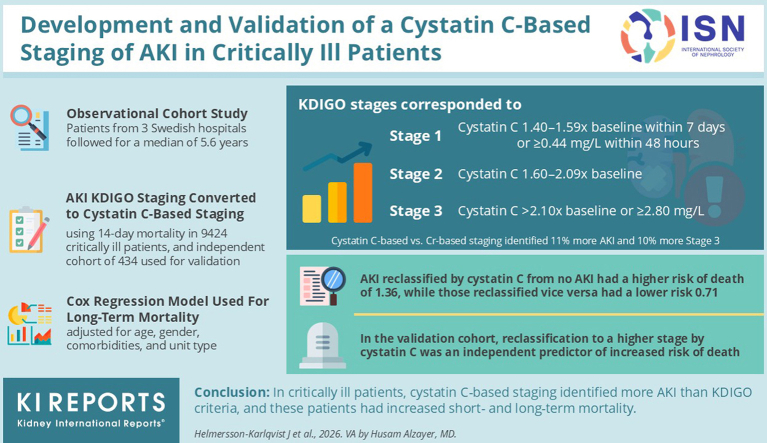

Acute dysfunction of the kidneys is a severe condition that is common in intensive care,^1^ mandating accurate and timely diagnosis with major therapeutic and prognostic implications. Moreover, uniform definitions of acute renal dysfunction are important to facilitate the comparison of patient populations to interpret and implement findings in different studies.

Although the kidneys have a variety of functions, estimating the GFR is the most widely used indication of renal function. The concept of AKI based on serum creatinine levels or urine output has been used in several definitions of AKI,^2^^,^^3^ the most recent being the KDIGO criteria.^4^ The estimation of the endogenous substance creatinine is the most commonly used method for estimating renal function, but its relation to GFR is limited by the assumption that the production and volume of distribution of creatinine are constant. However, creatinine-based estimated GFR (eGFR) in the intensive care unit (ICU) population has several challenges since the assumptions above are seldom fulfilled. Constant intake of meat products, steady state in GFR and fluid balance, and normal and constant muscle mass are rare in ICU patients. Additionally, urine output, although readily measured in ICU patients, is less frequently reported in large populations in a way that allows assessment of hourly output.

Cystatin C, another endogenous substance used for GFR assessment, has constant production independent of muscle mass and protein intake but may be influenced by cortisol levels and thyroid function.^5^^,^^6^ Cystatin C based eGFR calculation differs from that calculated from serum creatinine, especially in the ICU at higher actual GFR.^7^ Recent reports suggest that eGFR with cystatin C corresponds better to the gold standard of GFR measurement, iohexol clearance, than eGFR with creatinine in critically ill patients.^8^ Additionally, an increasing bulk of evidence suggests that serum cystatin C estimates GFR,^9^ and predicts the risk of death better than creatinine does in the general population.^10^ Recent data also suggest equal or better performance of serum cystatin C compared with creatinine as predictor of ICU outcomes in ICU patients.^11^, ^12^, ^13^

Given its more advantageous profile to estimate GFR, especially at mild to moderate decrease in GFR, identifying patients with AKI with cystatin C may be useful since this could alert the clinician earlier than with creatinine to consider actions for prevention or treatment of AKI. We hypothesized that serum or plasma cystatin C-derived eGFR levels corresponding to current AKI stages would be a better predictor of outcome than creatinine-based AKI staging. Accordingly, we set out to establish a cystatin C-based AKI staging and assess its performance in a large Swedish cohort of critically ill patients using a comprehensive discovery-validation cohort approach.

## Methods

This multicenter observational cohort study was approved by the Swedish Ethical Review Authority (Dnr 2013/441). The Helsinki Declaration and its subsequent revisions were followed. The study is reported according to the Strengthening the Reporting of Observational Studies in Epidemiology checklist.

### Discovery Cohort

The discovery cohort consisted of ICU patients from Uppsala, Karolinska, and Lund University Hospitals from 2006 to 2013, who had both plasma creatinine and cystatin C simultaneously analyzed for at least 7 days. The samples were collected in the same tube and immediately analyzed at Uppsala, Karolinska, and Lund University Hospital Laboratories, respectively. Adult patients ≥ 18 years, with a complete personal identity number, were included. Valid results of creatinine, cystatin C, age, gender, and sampling date were retrieved from the laboratory information systems, and only the first series of measurements for each individual were included. Baseline creatinine and cystatin C levels were defined as the first values during the admission.

**Figure 1:**
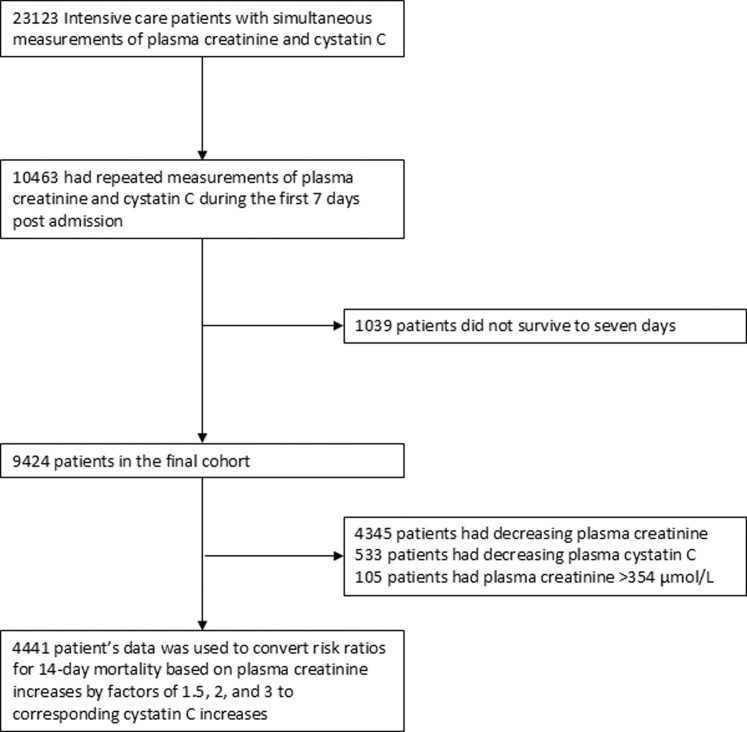
Flowchart of patient selection.

There were 23,123 unique patients with simultaneous measurements of plasma creatinine and cystatin C (Figure 1). Of these, 10,463 had repeated measurements during the first 7 days postadmission, but 1039 patients did not survive to 7 days. Consequently, the final cohort was made up of 9424 patients.

To find corresponding changes in plasma creatinine and cystatin C, we calculated creatinine and cystatin C ratios between the maximal value within 7 days and the first (baseline) value. Since the population was defined by the availability of plasma creatinine and cystatin C, no patients were lost to follow up, and there were no missing data in the study.

### Validation Cohort

The DEMONSTRATE study was approved by the Swedish Ethical Review Authority (approval numbers 2022-01051-01, 2022-06306-02, and 2024-04959-02). Given the nature of the study, informed consent was waived.

Data were extracted from clinical chemistry departments in 6 mid-Sweden counties (Uppsala, Gävleborg, Västmanland, Sörmland, Värmland, and Örebro), collectively representing approximately 18% of Sweden’s population (1.86 million residents). Data were also extracted from the National Patient Register and the Cause of Death Register. The data from each source were linked for each patient using the personal identification number, further pseudonymized, and compiled to form the DEMONSTRATE database. The validation cohort consisted of 2124 patients with both plasma creatinine and cystatin C, whereof 434 simultaneously analyzed within 7 days from hospital admission between 2016 and 2022.

### Measurement of Creatinine, Cystatin C, and Estimation of eGFR

Plasma creatinine in μmol/l was analyzed with isotope dilution mass spectrometry-calibrated methods as follows: at Uppsala Hospital Laboratory a modified kinetic Jaffe method from 2004 to 2008 and an enzymatic method from 2009 to 2015 on Architect ci8200 (Abbott Laboratories, Abbott Park, IL,; at Karolinska University Laboratory with a modified kinetic Jaffe method on UniCel DXC800 (Beckman Coulter, Brea, CA); and at Lund hospital laboratory with an enzymatic method on Roche Cobas c501 (Roche Diagnostics, Rotkreuz, Switzerland). All laboratories used nationally standardized, isotope dilution mass spectrometry-traceable calibration throughout. Plasma cystatin C in mg/l was analyzed with an assay from Dade Behring on a BN ProSpec analyzer (Siemens Healthcare Diagnostics, Solna, Sweden) at Uppsala and Karolinska until 2007 and thereafter with an assay from Gentian with similar calibration (Gentian, Moss, Norway) on Architect ci8200 (Uppsala) and UniCel DXC800 (Karolinska). Cystatin C was analyzed with a BN ProSpec analyzer and later with reagents from Roche on Roche Cobas c501 in Lund. The assays from Gentian and Roche were traceable to the international calibrator ERM-DA471/IFCC.^14^^,^^15^ All the 3 hospital laboratories were accredited and participated in external quality assurance programs (Equalis.se).

eGFR_Cr_ was calculated using the Chronic Kidney Disease-Epidemiology Collaboration creatinine equation from 2009 without adjustment for race.^16^ eGFR_Cyst_ was calculated from plasma cystatin C using the International Federation of Clinical Chemistry and Laboratory Medicine-equation Caucasian, Asian, pediatric, and adult cohorts.^17^

### Comorbidities and Outcomes

Comorbidity data from September 6, 1999 were retrieved from the Swedish National Patient Register, which records data, including diagnoses, from all inpatient hospital visits in Sweden.^18^ The majority of the included patients had been admitted to hospital care before the studied intensive care visit. Only 3510 patients (16%) had no previous hospital care recorded in the National Patient Register. Charlson comorbidity index was calculated as a measure of chronic illness burden.^17^ Mortality data was retrieved from the Swedish Cause of Death Register, covering data from all Swedish residents.^19^ The Swedish National Board of Health and Welfare administers both registers.

### Calculations and Statistical Analyses

Data are presented as n (percentage), median (interquartile range), mean (SD), or hazard ratio (HR; 95% confidence interval, [CI]) as appropriate. Model development in the discovery cohort is detailed in the Supplementary material.

We chose the 14-day mortality arbitrarily to mirror early AKI-related mortality as an end point to find corresponding increases and absolute changes in plasma creatinine and cystatin C. First, risk ratios were calculated in a log-binomial model with 14-day mortality as an end point and ratios between maximal and admission creatinine and cystatin C as restricted cubic splines. In this calculation, patients with baseline plasma creatinine levels above 354 μmol/l were removed from the analysis, as were patients with plasma creatinine and cystatin C levels decreasing from the baseline. Mortality was calculated from the day after 7 days of simultaneous measurements of plasma creatinine and cystatin C. Briefly, based on risk ratios of 14-day mortality the following were calculated at: (i) creatinine ratios 1.5, 2, and 3 were translated to cystatin C ratios; (ii) creatinine concentration of 353.6 μmol/l was translated to cystatin C concentration; and (iii) a creatinine increase of 26.5 μmol/l over 48 hours translated to the corresponding cystatin C increase defined by the KDIGO criteria.^4^ A more detailed description of the model development can be found in the Supplementary Methods section of the Supplementary File.

In the discovery cohort, patients were staged for AKI according to KDIGO creatinine criteria and the corresponding cystatin C classification that was developed in the present study. Mortality rate (per 100 person-years at risk) during follow-up for each AKI stage for plasma creatinine and cystatin C was calculated, and the association using AKI stage 0 as reference was estimated with Cox proportional hazards models. In these analyses, follow-up time was accrued between day 8 after admission to the ICU till the date of death or the end of follow-up, December 30, 2016. Cox models were adjusted for age, gender, Charlson comorbidity index, and the type of ICU. Reclassification of patients to higher or lower AKI stages based on cystatin C was assessed for HR of mortality compared with nonreclassified patients. A sensitivity survival analysis was performed with adjustment for site as a random effect.

A sensitivity analysis of reclassifications was performed on subcohorts of patients with and without infectious diseases, considering the potential impact of steroid treatment, common in these patients, on cystatin C levels. In order to adjust for underlying kidney dysfunction, we performed a sensitivity analysis of the role of reclassification with baseline creatinine as a covariate.

To quantify improvement in 365-day mortality prediction after adding the cystatin C-based AKI definition, we calculated the continuous net reclassification improvement (NRI) and the integrated discrimination improvement. Predicted probabilities from the creatinine-based model and the cystatin C model were compared. Continuous NRI assessed whether predicted risk increased among cases and decreased among noncases. The integrated discrimination improvement quantified the change in average predicted risk separation between cases and noncases. CI were obtained via nonparametric bootstrapping.

To validate the usefulness of our developed cystatin C-based AKI classification, the added risk of reclassification according to cystatin C was assessed using Cox proportional hazards models adjusted for gender and age in both the development cohort and in an independent validation cohort. The follow-up time was accrued between day 8 after admission to the ICU until the date of death or the end of follow-up, 31 December, 2024. Reclassification was defined as follows:

$$\text{AKI}\;{\text{stage}}_{\left(\text{cystatin}\;C\right)}\;–\;\text{AKI}\;{\text{stage}}_{\left(\text{creatinine}\right)}\text{.}$$

The performance of cystatin C and creatinine-based AKI staging for mortality was compared using Akaike’s information criterion.

STATA (version 16, StataCorp LLC) and R (version 4.2.3, Posit PBC) were used for calculations.

## REsults

### The Discovery Cohort

The median follow-up of the 9424 patients was 5.6 (2.8–7.2) years. The characteristics of the cohort are presented in Table 1. The cohort consisted of both medical and surgical patients, where the majority were males, and most patients had some comorbidities. Infectious and cardiovascular diseases were the most common discharge diagnoses.

**Table 1:** Baseline characteristics of the 9424 included study participants in the developmental cohort

Characteristics	Mean (SD)
Age, yrs	62 (16)
Charlson comorbidity index	1.8 (2.2)
eGFR, creatinine CKD-EPI, ml per 1.73 m^2^	72 (34)
eGFR, cystatin C CAPA, ml per 1.73 m^2^	66 (39)

	*n* (%)
Female gender	3292 (34.9%)
Type of intensive care unit	
General intensive care unit	5510 (58.5%)
Neurosurgical intensive care unit	16 (0.17%)
Cardiothoracic intensive care unit	3941 (41.8%)
Coronary care unit	30 (0.32%)
Total mortality during the follow-up	3524 (37.4%)
Total mortality within 30 d	601 (6.4%)
Discharge diagnoses^a^	
Diabetes mellitus	1686 (17.9%)
Obesity	209 (2.2%)
Hypertension	2741 (29.1%)
Cardiovascular disease	4182 (44.4%)
Infections	2889 (30.5%)
Kidney disease	1201 (12.7%)
Trauma	1169 (12.4%)
Cerebrovascular disease	500 (5.3%)
Liver and biliary tract disease	492 (5.2%)
Intoxications	66 (0.70%)

CAPA, Caucasian, Asian, pediatric, and adult^17^; CKD-EPI, Chronic Kidney Disease Epidemiology Collaboration; eGFR, estimated glomerular filtration rate.^16^

a Patients can have several discharge diagnoses.

### Cystatin C-Based AKI Staging

To convert relevant creatinine measures into corresponding cystatin C measures, we used a log-binomial model using restricted cubic splines to calculate risk ratios for 14-day mortality as described in detail in the Methods and the Supplements. Risk ratios for 14-day mortality for plasma creatinine increase by factors 1.5, 2, and 3, also used as knots, were converted for similar risk ratios for 14-day mortality for cystatin C at 1.60, 2.45 and 3.41, and corresponding ratios between maximal and admission cystatin C 1.4, 1.6, and 2.1 respectively (Supplementary Figure S1). Secondly, at an increase to plasma creatinine of 354 μmol/l, i.e., the absolute limit of stage 3 AKI,^4^ the risk ratio for death within 14 days was 3.21, and the same risk ratio for cystatin C was at 2.80 mg/l (Supplementary Figure S2). Creatinine 95 μmol/l and cystatin C 1.34 mg/l, i.e., at the upper limit of the reference interval of a middle-aged population of both genders, were used as reference.^20^ Finally, an absolute creatinine increase of 26.5 μmol/l within 48 hours had a risk ratio for death within 14 days of 1.51, corresponding to an increase in cystatin C with 0.44 mg/l within the same period (Supplementary Figure S3).

Based on these results, the KDIGO AKI stages corresponded to the following increases in plasma cystatin C:

- Stage 1: Increase in cystatin C 1.40–1.59 times baseline within 7 days, or increase in plasma cystatin C by ≥ 0.44 mg/l within 48 hours
- Stage 2: Cystatin C 1.60–2.09 times baseline
- Stage 3: Cystatin C >2.10 times baseline OR increase in plasma cystatin C to ≥ 2.80 mg/l.

### Association of Creatinine-Based and Cystatin C-Based AKI With Mortality

**Figure 2:**
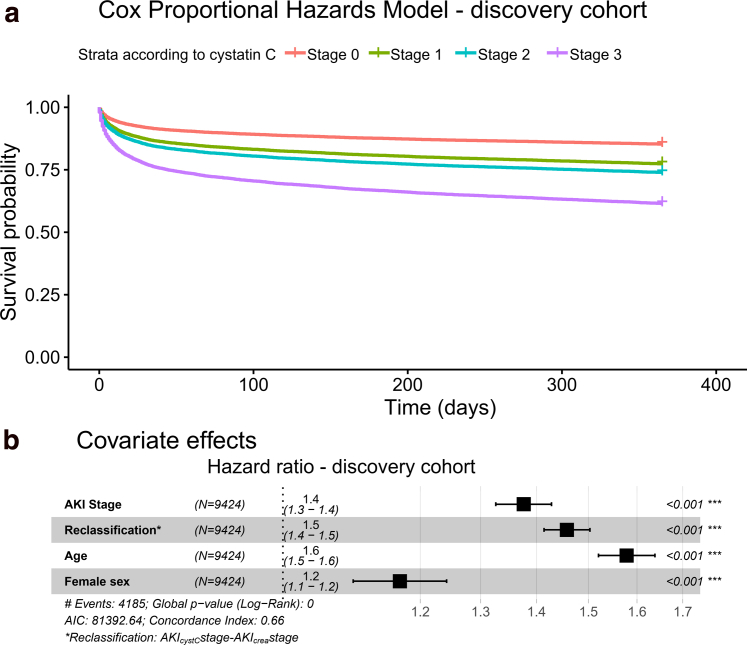
Cox proportional hazards model for mortality during follow-up with the added risk of reclassified according to cystatin C adjusted for gender and age in the discovery cohort depicted for AKI stages (a) and covariates (b). AKI, acute kidney injury; AIC, Akaike’s information criterion.

For both creatinine and cystatin C-based AKI, the crude mortality rate increased with increasing AKI stage (Table 2). Increasing AKI stage according to cystatin C was associated with a higher risk of death during the follow-up (Figure 2a). For creatinine-based AKI, the adjusted HR (95% CI) for mortality was highest for stage 2 as follows: 1.7 (1.4–2.0). For the cystatin C-based AKI, the highest HR was observed for AKI stage 3 as follows: 1.9 (1.8–2.0).

**Table 2:** Incidence rates and total mortality estimates in AKI-stages based on creatinine or cystatin C using Cox regression models

	AKI stage	n at risk/n of events	Incidence rate, failures/100 person-years (95% CI)	Hazard ratio (95% CI)	Hazard ratio, adjusted^a^ (95% CI)
Based on Creatinine	0	6773/2254	6.3 (6.0–6.5)	Ref	Ref
	1	1685/726	8.8 (8.2–9.5)	1.4 (1.3–1.5)	1.2 (1.1–1.3)
	2	262/129	11.7 (9.8–13.9)	1.8 (1.5–2.1)	1.7 (1.4–2.0)
	3	704/414	14.3 (13.0–15.8)	2.1 (1.9–2.4)	1.3 (1.2–1.4)
Based on Cystatin C	0	5763/1723	5.4 (5.2–5.7)	Ref	Ref
	1	1362/515	7.4 (6.8–8.1)	1.4 (1.2–1.5)	1.1 (1.0–1.3)
	2	613/211	6.7 (5.9–7.7)	1.2 (1.1–1.4)	1.1 (1.0–1.3)
	3	1686/1074	16.8 (15.8–17.8)	3.0 (2.8–3.2)	1.9 (1.8–2.0)

AKI, acute kidney injury; CI, confidence interval.

a Adjusted for age, gender, Charlson comorbidity index, and type of intensive care unit.

### Reclassification With AKI Classification According to Cystatin C

With AKI classification according to cystatin C, 424 patients were reclassified to lower AKI stages and 2333 patients were reclassified to higher AKI stages compared with classification according to creatinine (Table 3). Reclassification to a higher AKI stage according to cystatin C from AKI stage 0 based on creatinine conferred a higher HR of mortality; HR: 1.36 (95% CI: 1.24–1.49). For reclassification form creatinine-based AKI stages 1 and 2, the corresponding HRs were 1.09 and 1.39, respectively, with lower precision. Reclassification from a higher creatinine-based AKI stage to a lower cystatin C-based AKI stage was associated with lower HR of mortality; HR (95% CI) for reclassification to creatinine-based AKI stage 1 was as follows: 0.71 (0.56–0.91).

**Table 3:** Total mortality risk when AKI is classified according to cystatin C, as compared with creatinine

AKI-staging by creatinine	Evaluated (N)	Reclassified to lower AKI-stage by cystatin C	Not reclassified (same AKI-stage)	Reclassified to higher AKI-stage by cystatin C
		HR (95 % CI)	HR (95 % CI)	HR (95 % CI)
	6773		5377 (1593 events)	1396 (661 events)
AKI 0			Ref	1.87 (1.71–2.05)^a^
AKI 0, adj			Ref	1.36 (1.24–1.49)^a^
	1685	337 (104 events)	575 (227 events)	773 (395 events)
AKI 1		0.73 (0.57–0.91)^a^	Ref	1.42 (1.20–1.67)^a^
AKI 1, adj		0.71 (0.56–0.91)^a^	Ref	1.09 (0.92–1.30)
	262	21 (7 events)	77 (27 events)	164 (95 events)
AKI 2		0.87 (0.38–2.00)	Ref	2.00 (1.31–3.08)^a^
AKI 2, adj		0.62 (0.27–1.43)	Ref	1.39 (0.88–2.18)
	704	66 (32 events)	638 (382 events)	
AKI 3		0.75 (0.52–1.08)	ref	
AKI 3, adj		1.21 (0.83–1.74)	ref	

adj, adjusted for age, gender, CCI and type of intensive care unit; AKI, acute kidney injury; CI, confidence interval; HR, hazards ratio.

a The result is statistically significant at α = 0.05.

Reclassification for all stages adjusted for gender and age showed a clear effect of AKI stage defined using creatinine (HR: 1.4 [95% CI: 1.3–1.4], *P* < 0.001), and increased mortality in patients reclassified to a higher AKI stage using cystatin-C (HR: 1.5 [95% CI: 1.4–1.5], *P* < 0.001; Figure 2b). A sensitivity survival analysis was performed with adjustment for site as a random effect, which showed similar effect sizes as the main analysis, but the effect of gender was no longer significant (Supplementary Table S1).

The continuous NRI comparing a cystatin C-based logistic model with a creatinine model was 0.35, indicating overall improvement in classification. However, this was driven by a substantial improvement in correctly reducing risk estimates among nonevents (NRI^-^ = 0.73), whereas classification of events worsened (NRI^+^ = -0.38).

The integrated discrimination improvement comparing the cystatin C model with the creatinine model was 0.026 (95% CI: 0.023–0.029), indicating a statistically significant improvement in discrimination.

### Additional Calculations

The findings were similar for 30-day mortality as the end point (Supplementary Table S2). A sensitivity analysis was performed in the subcohort of patients with and without infectious diseases as discharge diagnoses, since steroid treatment may affect cystatin C levels^21^ and critically ill patients with sepsis may have been treated with steroids.^22^ The findings in infected patients were similar to those in the whole cohort, although some subgroups had very few events (Supplementary Tables S3–S6).

### Validation

**Figure 3:**
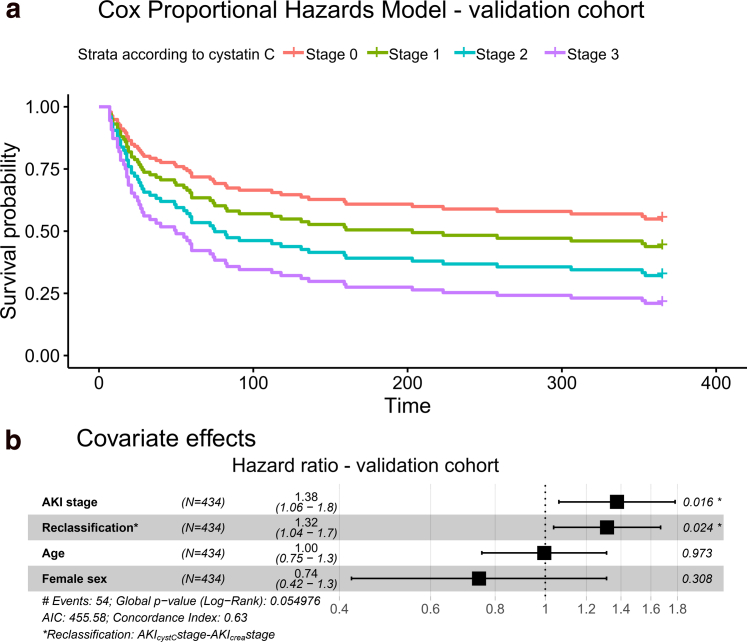
Cox proportional hazards model for mortality during follow-up with the added risk of reclassified according to cystatin C adjusted for gender and age in the validation cohort depicted for AKI stages (a) and covariates (b). AKI, acute kidney injury; AIC, Akaike’s information criterion.

Validation was performed in an independent cohort of 434 patients for whom an AKI stage could be calculated using both creatinine and cystatin C during their first hospital admission. Their baseline characteristics are presented in Supplementary Table S7. A Cox proportional hazards model adjusted for gender and age showed a clear effect of AKI stage defined using creatinine (HR: 1.38 [95% CI: 1.06–1.8], *P* = 0.016; Figure 3a), and increased mortality in patients reclassified to a higher AKI stage using cystatin-C (HR: 1.32 [95% CI: 1.04–1.7], *P* = 0.024; Figure 3b). A model including Charlson comorbidity index as an additional sensitivity analysis was performed and produced the same estimates because of low comorbid burden in the cohort with complete AKI data. The effect of reclassification remained in the sensitivity analysis, where we included baseline kidney dysfunction as a covariate (Supplementary Table S8). Short-term mortality could not be analyzed using the multivariable model in the validation cohort because of low mortality rate at 30-days.

### Performance

Cystatin C performs better in predicting mortality during follow-up both in the discovery cohort and the validation cohort, according to Akaike’s information criterion (Akaike’s information criterion: discovery cystatin C = 81397, creatinine = 81882, and validation cystatin C = 454, creatinine = 459).

## Discussion

### Key Findings

In a large, multicenter cohort of ICU patients, cystatin C-based AKI staging identified 11% more AKI cases and 10% more cases with stage 3 AKI than AKI staging according to creatinine. Patients reclassified to AKI according to cystatin C from no AKI according to creatinine had an increased risk of death, whereas patients reclassified vice versa had a decreased risk of death. Patients classified as stage 3 AKI according to cystatin C had an increased risk of death compared with no AKI irrespective of AKI staging according to creatinine. These findings were consistent for 30-day mortality and in patients with and without infections. Finally, in an independent validation cohort, reclassification to AKI according to cystatin C versus creatinine-based staging was an independent predictor of the risk of death.

We report that a cystatin C-based system to detect AKI is more sensitive in finding patients with AKI than the current standard classification system, where creatinine is used for the same purpose. Detection of AKI is important to identify patients with significant deterioration in renal function and to trigger medical interventions to counteract further deterioration and potentially reverse this. The endogenous renal markers creatinine and cystatin C can be used to monitor renal function in steady-state conditions. However, monitoring renal function in the dynamic conditions of critical illness is challenging since renal function can deteriorate within hours. The rationale for using Cystatin C is that it can be measured on standard laboratory instruments with low turnaround times, and has been reported to estimate GFR more accurately than creatinine when compared with the gold standard, iohexol clearance, under the dynamic conditions of cardiac surgery.^9^ Accordingly, GFR markers that are accurate and sensitive would probably lead to earlier detection of AKI. In addition to identifying an increased number of AKI cases when using the suggested cystatin C staging, we also found that patients reclassified from not having AKI according to creatinine also had a higher risk of death, indicating a valid reclassification. Conversely, patients having AKI according to creatinine but not according to cystatin C had a lower risk of death. One potential reason for the more cases detected is that the molecular weight of cystatin C is more than 100 times larger than that of creatinine, and a potential pathophysiological process for AKI with decreasing pore size in the glomeruli would potentially lead to limited clearance for larger molecules initially. This concept, commonly referred to as shrunken pore syndrome, has been described in intensive care patients and is associated with increased mortality,^23^^,^^24^ which could account for part of our findings. Additionally, low muscle mass may blunt creatinine changes that reflect renal function, potentially contributing to the superior performance of cystatin C as a mortality predictor. However, the present study could not assess the effects of muscle mass because of lack of available data.

The risk of death was increased in patients with AKI according to cystatin C but not according to creatinine. This was seen both at 30 days and long-term follow-up, more than 5 years on average. Correspondingly, cystatin C but not creatinine was a predictor of chronic kidney disease and long-term mortality in a recent report on patients followed up after AKI stage 3 after ICU.^25^

Finding the risk of death with cystatin C corresponding to different AKI criteria according to KDIGO was performed using mortality at 14 days after admission. The rationale for this was to assess mortality shortly after AKI criteria were fulfilled, which can take up to 7 days. Assessment of mortality earlier would shorten the time to develop complications for renal dysfunction, whereas assessing later from admission would have been influenced more by chronic illnesses.^26^

Another factor that may influence our findings is steroid treatment, which may have an impact on the production of cystatin C.^27^, ^28^, ^29^ Hydrocortisone was commonly used for the treatment of septic shock during the study,^30^ and consequently, we performed a posthoc analysis in patients with infections. Although some power was lost when analyzing patients with infections constituting less than one-third of the cohort, the results seen in the whole cohort were similar in these patients.

### Strengths and Limitations

This is, as far as we know, the first study suggesting a staging for AKI based on cystatin C. The study was performed in a large cohort of over 9000 patients. Moreover, patient follow-up was over 5 years, and no patients were lost to follow-up. Finally, the cystatin C-based AKI staging model was evaluated in an independent cohort of patients, confirming its performance.

The study also has limitations. As in many epidemiological studies, diuresis was not used for the AKI definition because of a lack of data. However, since the study aimed to evaluate endogenous biomarkers of renal dysfunction, i.e., cystatin C versus creatinine, the study provides new insights. The baseline creatinine and cystatin C values used may not always reflect the patients’ habitual levels. However, many patients may lack habitual measurements, particularly for both creatinine and cystatin C. In the creatinine to cystatin C conversion model, we excluded patients with plasma creatinine levels >354 μmol/l and those with a decrease in plasma creatinine and cystatin C from baseline. Although we cannot rule out that some of these patients experienced AKI at admission that resolved during hospitalization, the cystatin C based AKI model was robust in the final study cohort. We did not have a renal outcome as an endpoint because of the lack of data on chronic renal failure and chronic renal replacement therapy. However, mortality is a robust outcome that at least in part reflects severe renal illness and was also used to validate KDIGO AKI definitions. We selected 14-day mortality *a priori* to capture outcomes most directly related to the acute illness and AKI-associated risk, although this choice may limit comparability with studies using 30-day mortality. Another limitation is that the mortality risk did not separate clearly between AKI stages 1 and 2, likely reflecting the relatively small number of patients in stage 2. However, a significant ordinal trend across stages 0 to 3 was evident, and the reclassification analyses showed consistently higher risk when patients were upstaged by cystatin C and lower risk when downstaged, reinforcing the robustness of the findings. We do not report racial or socioeconomic demographic variables, as these data are not recorded in Swedish clinical registries and cannot legally be collected as part of routine healthcare documentation. Also, potential changes in ICU practice over time could not be fully assessed and represent a limitation of the study. Finally, when modeling risk ratios using splines, the exact estimates are sensitive to the data distribution and the placement of spline knots, particularly since knots are set at percentiles, except in the initial model, which is most influential. Nevertheless, the approach remains valuable as it captures the overall relationship between the predictor and mortality.

### Clinical Implications

These results showed that AKI staging according to cystatin C will identify more patients with AKI and those with an increased risk of death, suggesting that the cystatin C-based AKI definition is clinically relevant. Future studies need to validate the cystatin C-based AKI staging in other cohorts and also on long-term renal outcomes.

## Conclusion

In a large cohort of critically ill patients, we developed a cystatin C-based staging for AKI corresponding to creatinine-based AKI staging. The cystatin C-based staging identified more patients with AKI and those with an increased risk of short and long-term death.

## Disclosure

JÄ has received lecturing fees from AstraZeneca, Novartis, and Boehringer Ingelheim and served on advisory boards for AstraZeneca, Boehringer Ingelheim, and Astella, all unrelated to the present paper. All other authors declare that they have no conflicts of interest.

## Supplementary Material

Supplementary file (PDF) **Supplementary Methods**. **Figure S1.** Risk ratio of 14-day mortality for creatinine ratio (left) and cystatin C ratio (right). **Figure S2.** Risk ratio of 14-day mortality based on the first creatinine value. Figure S3. Risk ratio of 14-day mortality by maximum increase in creatinine (left) and cystatin C (right) over 48 hrs. **Table S1.** Cox proportional hazards model for mortality during follow-up with adjustment for ICU type as a random effect. **Table S2.** Thirty-day mortality risk when AKI is classified according to cystatin C, as compared to creatinine. **Table S3.** Total mortality risk as hazard ratios (95% confidence intervals) across AKI. categories when classified according to creatinine or cystatin C in patients without infections. The model is adjusted for age, gender, CCI and type of intensive care unit. **Table S4.** Total mortality risk as hazard ratios (95% confidence intervals) across AKI categories when classified according to creatinine or cystatin C in patients with infections. The model is adjusted for age, gender, CCI, and type of intensive care unit. **Table S5.** Total mortality risk as in hazard ratios (95% confidence intervals) when AKI is classified according to cystatin C, as compared with creatinine in the sub-cohort of patients without infections. **Table S6.** Total mortality risk as in hazard ratios (95% confidence intervals) when AKI is classified according to cystatin C, as compared with creatinine in the sub-cohort of patients with infections. **Table S7.** Baseline characteristics of the 434 included in the validation cohort. **Table S8.** Cox proportional hazards model for mortality during follow-up when AKI is classified according to cystatin C in the validation cohort. The first creatinine after admission was included in the model. TRIPOD Checklist.
