# Supplementary material for: Development and Validation of a Cystatin C-based Staging of AKI in Critically Ill Patients
Source: Kidney Int Rep. 2026 Mar 25;11(6):106514. doi: 10.1016/j.ekir.2026.106514 (PMC13101441; doi:10.1016/j.ekir.2026.106514)
Supplement: Supplementary file (PDF) — Supplementary Methods. Figure S1. Risk ratio of 14-day mortality for creatinine ratio (left) and cystatin C ratio (right). Figure S2. Risk ratio of 14-day mortality based on the first creatinine value. Figure S3. Risk ratio of 14-day mortality by maximum increase in creatinine (left) and cystatin C (right) over 48 hrs. Table S1. Cox proportional hazards model for mortality during follow-up with adjustment for ICU type as a random effect. Table S2. Thirty-day mortality risk when AKI is classified according to cystatin C, as compared to creatinine. Table S3. Total mortality risk as hazard ratios (95% confidence intervals) across AKI. categories when classified according to creatinine or cystatin C in patients without infections. The model is adjusted for age, gender, CCI and type of intensive care unit. Table S4. Total mortality risk as hazard ratios (95% confidence intervals) across AKI categories when classified according to creatinine or cystatin C in patients with infections. The model is adjusted for age, gender, CCI, and type of intensive care unit. Table S5. Total mortality risk as in hazard ratios (95% confidence intervals) when AKI is classified according to cystatin C, as compared with creatinine in the sub-cohort of patients without infections. Table S6. Total mortality risk as in hazard ratios (95% confidence intervals) when AKI is classified according to cystatin C, as compared with creatinine in the sub-cohort of patients with infections. Table S7. Baseline characteristics of the 434 included in the validation cohort. Table S8. Cox proportional hazards model for mortality during follow-up when AKI is classified according to cystatin C in the validation cohort. The first creatinine after admission was included in the model. TRIPOD Checklist. [file mmc1.pdf]

Supplemental material - Development and validation of a Cystatin C-based staging of acute kidney injury in critically ill patients

Supplementary Methods – The Development of the model

Simultaneously measured creatinine and cystatin C from patients in ICU in Stockholm, Uppsala, and Lund from 2006-2013, and mortality data from the National Board of Health and Welfare from 06-09-1999 to 30-12-2016. The original number of unique patients was 23,123, of which 10,463 had repeated data during the first 7 days of hospitalization for creatinine and cystatin C and could be evaluated for changes. 9,424 were over 18 years old and were alive for 7 days after the first day in ICU.

Translation of creatinine thresholds for AKI to cystatin C via risk ratios:

1. Creatinine ratios 1.5, 2, and 3 translated to cystatin C ratios.
2. Creatinine 353.6 µmol/L translated to cystatin C value.
3. Creatinine increase of 26.5 µmol/L over 48 hours translated to cystatin C increase.

1. Creatinine ratios 1.5, 2, and 3 translated to cystatin C ratios

Creatinine ratio and cystatin C ratio were calculated as the highest value during hospitalization divided by the first value (baseline) within a maximum of 7 days. Creatinine ratios 1.5, 2, and 3 were translated to cystatin C ratios.

Baseline creatinine values >353.6 (first value) were removed, in this part, as they seemed to disrupt the relationship with decreasing risk ratios, possibly because these patients were treated more intensively; patients with high values of cystatin C were not removed.

All creatinine and cystatin C ratios <1 were removed, i.e., where the value decreased during hospitalization. The original creatinine-based AKI definition was not defined for values below 1, and the medical relationship was uncertain, moreover, it was unlikely that the risk of death decreased linearly if creatinine or cystatin C decreased.

The model was adjusted for baseline creatinine (when creatinine ratios were calculated) and baseline cystatin C (when cystatin C ratios were calculated), which seemed reasonable.

Spline curve with risk ratios that were calculated using a log-binomial model using the below code in Stata version 16.

mkspline splinekrea = kvotkrea, knots (1.5, 2, 3) cubic displayknots

mat knots = r(knots)

glm mort14 splinekrea kreafirs, fam(bin) link(log) nolog eform /*adjusted for baseline creatinine*/*

mkspline splinecyst = kvotcyst, cubic displayknots

mat knots = r(knots)

glm mort14 splinecyst cystati8, fam(bin) link(log) nolog eform /*adjusted for baseline cystatin C*/*

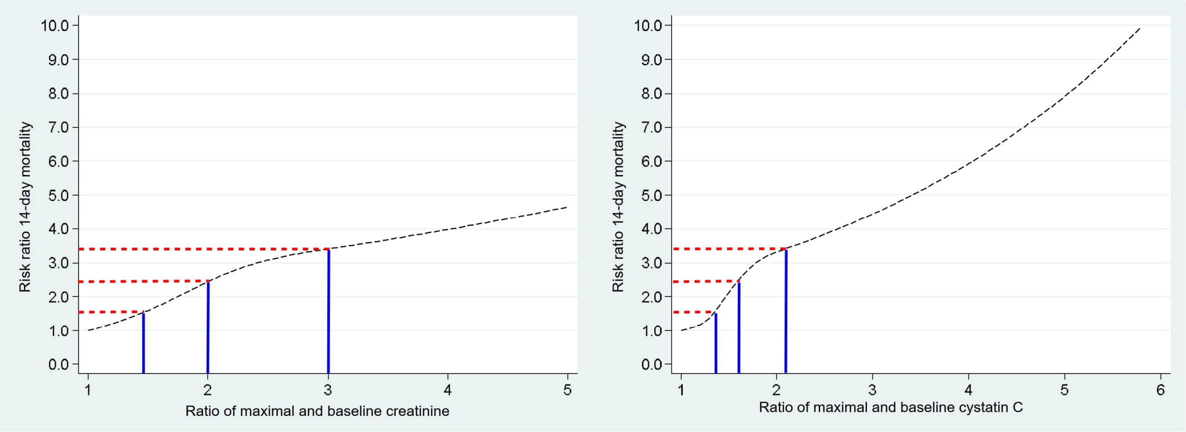

Supplemental Figure 1. Risk ratio of 14-day mortality for creatinine ratio (left) and cystatin C ratio (right), modelled as restricted cubic splines with three knots at predefined values of creatinine 1.5, 2, and 3 and five knots according to Harrell (Harrell, F. E., Jr. 2001. Regression Modeling Strategies: With Applications to Linear Models, Logistic Regression, and Survival Analysis. New York: Springer) at cystatin C ratios 1.02, 1.13, 1.25, 1.43, 2.08. The reference value is no change in creatinine i.e., a ratio of 1. Blue lines represent creatinine and cystatin C levels, while red lines indicate corresponding risk ratios.

Creatinine ratios 1.5, 2, and 3 corresponded to risk ratios 1.60, 2.45, and 3.41 (left). These risk ratios correspond to cystatin C ratios 1.4, 1.6, and 2.1 (right).

2. Creatinine 353.6 µmol/L translated to cystatin C value

The chosen reference point at the upper limit of "normal" creatinine was set at 95 µmol/L, since according to Shlipak et al. (Shlipak MG, Matsushita K, Arnlov J, Inker LA, Katz R, Polkinghorne KR, Rothenbacher D, Sarnak MJ, Astor BC, Coresh J, et al: Cystatin C versus creatinine in determining risk based on kidney function. N Engl J Med. 2013;369:932-943) the reference interval is 45-90 µmol/L for women and 60-105 µmol/L for men.

The chosen reference point was cystatin C 1.34 mg/L as the average age in the cohort was 62 years. The reference interval for cystatin C was 0.72-1.34 mg/L for 60-69 years and 0.75-1.44 for >70 years.

A log-binomial model using restricted cubic splines with five knots was used to calculate risk ratios, using the above specified reference values and the below code in Stata:

mkspline splinekrea = kreafirs, cubic displayknots

mat knots = r(knots)

glm mort14 splinekrea, fam(bin) link(log) nolog eform*

Knot locations were based on Harrell's recommended percentiles. Ref Harrell, F. E., Jr. 2001. Regression Modeling Strategies: With Applications to Linear Models, Logistic Regression, and Survival Analysis. New York: Springer.

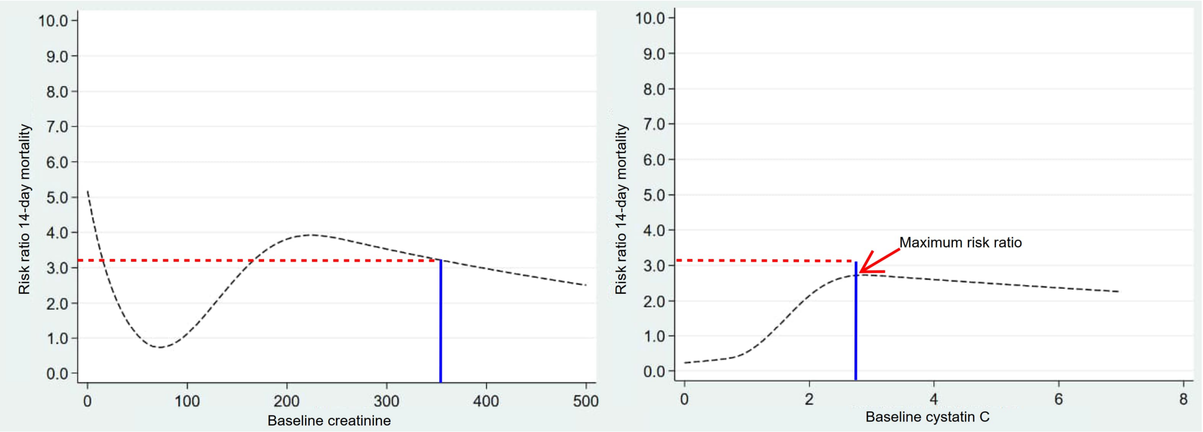

Knots (5) Knots (5)

Supplemental Figure 2. Risk ratio of 14-day mortality based on the first creatinine value. Creatinine 95 µmol/L and cystatin C 1.34 mg/L, i.e., at the upper limit of the reference interval of a middle-aged population of both gender, were used as reference. Knots were placed at 45, 66, 82, 107, 266 µmol/L for the first value of creatinine and at 0.57; 0.84; 1.06; 1.44; 3.18 mg/L for the first value of cystatin C. A creatinine concentration of 353.6 µmol/L (355) corresponded to a risk ratio 3.21 (left). For cystatin C, the highest risk ratio was 2.73, corresponding to a cystatin C concentration of 2.8 mg/L (right). Consequently, the risk ratio of 2.73 was used to determine the cystatin C level. Blue lines represent creatinine and cystatin C levels, while red lines indicate corresponding risk ratios.

3. Creatinine increase of 26.5 µmol/L over 48 hours translated to cystatin C increase

Creatinine increase of 26.5 µmol/L over 48 hours was translated to cystatin C increase over the same period using risk ratios modelled with a log-binomial model for 14-day mortality using restricted cubic splines with four knots, placed according to Harrell's recommendation, and using the below code in Stata. The reference value is no increase in creatinine i.e., 0. Four knots, rather than five, were chosen because of sample size limitations.

mkspline splinekrea = Maxkrea48h, cubic nknots(4)

mat knots = r(knots)

glm mort14 splinekrea kreafirs, fam(bin) link(log) nolog eform*

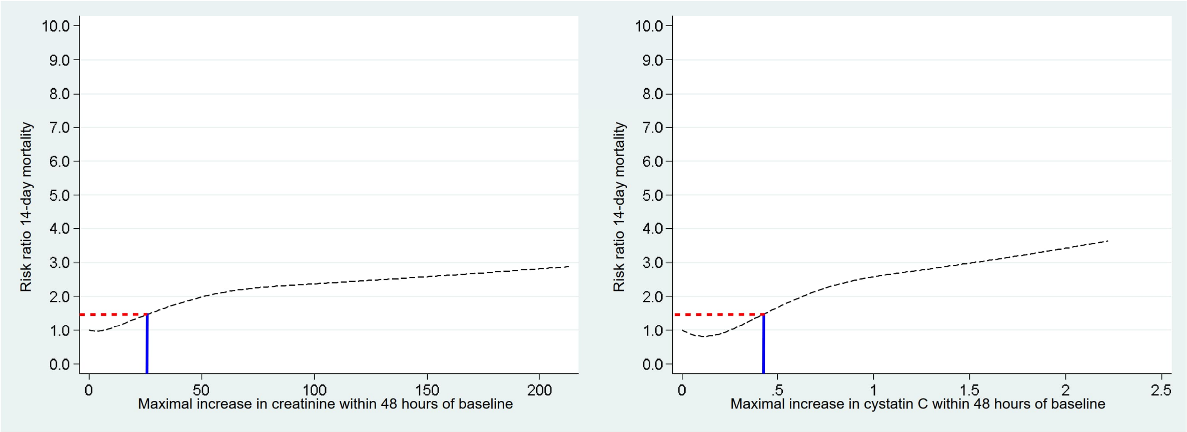

Knots (4) Knots (4)

Supplemental Figure 3. Risk ratio of 14-day mortality by maximum increase in creatinine (left) and cystatin C (right) over 48 hours. A maximum increase in creatinine over 48 hours of 26.5 µmol/L, which corresponded to a risk ratio of 14-day mortality of 1.51 (left). A risk ratio of 1.51 corresponded to a maximum cystatin C increase of 0.44 mg/L over 48 hours (right). Blue lines represent creatinine and cystatin C levels, while red lines indicate corresponding risk ratios.

Table S1. Thirty-day mortality risk when AKI is classified according to cystatin C, as compared to creatinine.

AKI-staging by creatinine	Evaluated (N)	Reclassified to lower AKI-stage by cystatin C<br>HR (95 % CI)	Not reclassified (same AKI-stage)<br>HR (95 % CI)	Reclassified to higher AKI-stage by cystatin C<br>HR (95 % CI)
	6773		5377 (203 events)	1396 (183 events)
AKI 0			ref	3.64 (2.98-4.44)
AKI 0, adj			ref	2.42 (1.98-2.96)
	1685	337 (12 events)	575 (39 events)	773 (100 events)
AKI 1		0.51 (0.27-0.98)	ref	1.96 (1.36-2.84)
AKI 1, adj		0.41 (0.21-0.78)	ref	1.17 (0.80-1.71)
	262	21 (1 events)	77 (9 events)	164 (43 events)
AKI 2		0.39 (0.05-3.09)	ref	2.44 (1.19-5.00)
AKI 2, adj		0.77 (0.03-2.14)	ref	1.64 (0.78-3.47)
	704	66 (9 events)	638 (67 events)	
AKI 3		1.34 (0.67-2.69)	ref	
AKI 3, adj		1.64 (0.80-3.35)	ref	

Adj = adjusted for age, gender, Charlson comorbidity index (CCI) and type of intensive care unit.

Table S2a. Total mortality risk as hazard ratios (95 % confidence intervals) across AKI categories when classified according to creatinine or cystatin C in patients without infections. The model is adjusted for age, gender, CCI and type of intensive care unit.

		AKI based on cystatin C			
		0	1	2	3
AKI based on creatinine	0	ref	1.03 (0.87–1.22)	1.10 (0.81–1.50)	1.94 (1.60–2.34)
	1	0.94 (0.73–1.21)	1.39 (1.17–1.66)	1.10 (0.85-1.41)	1.80 (1.51–2.15)
	2	N < 10	N < 10	1.35 (0.83–2.18)	2.14 (1.61–2.84)
	3	2.16 (1.25–3.74)	N < 10	N < 10	1.55 (1.33–1.81)

The number of patients in the table above:

		AKI based on cystatin C				
		0	1	2	3	Total
AKI based on creatinine	0	3,993	508	146	177	4,824
	1	245	446	205	253	1,149
	2	3	9	59	90	161
	3	21	8	4	377	410
	Total	4,262	971	414	897	6,544

Table S2b. Total mortality risk as hazard ratios (95 % confidence intervals) across AKI categories when classified according to creatinine or cystatin C in patients with infections. The model is adjusted for age, gender, CCI and type of intensive care unit.

		AKI based on cystatin C			
		0	1	2	3
AKI based on creatinine	0	ref	1.20 (0.99-1.46)	1.18 (0.86-1.61)	1.75 (1.47-2.08)
	1	0.94 (0.69-1.29)	1.24 (0.97-1.59)	0.86 (0.61-1.22)	1.41 (1.18-1.69)
	2	N < 10	N < 10	1.87 (1.00-3.50)	1.80 (1.33-2.45)
	3	0.99 (0.53-1.86)	N < 10	N < 10	1.03 (0.86-1.24)

The number of patients in the table above:

		AKI based on cystatin C				
		0	1	2	3	Total
AKI based on creatinine	0	1,384	248	92	225	1,949
	1	92	129	86	229	536
	2	4	5	18	74	101
	3	21	9	3	261	294
	Total	1,501	391	199	789	2,880

Table S3a. Total mortality risk asin hazard ratios (95 % confidence intervals) when AKI is classified according to cystatin C, as compared to creatinine in the sub-cohort of patients without infections.

AKI-staging by creatinine	Reclassified to lower AKI-stage by cystatin C<br>HR (95 % CI)	Not reclassified (same AKI-stage)<br>HR (95 % CI)	Reclassified to higher AKI-stage by cystatin C<br>HR (95 % CI)
		3993 (1003 events)	831 (324 events)
AKI 0		ref	1.71 (1.51-1.94)
AKI 0, adj A		ref	1.47 (1.29-1.66)
AKI 0, adj B		ref	1.26 (1.11-1.43)
	245 (63 events)	446 (154 events)	458 (212 events)
AKI 1	0.69 (0.51-0.92)	ref	1.48 (1.20-1.82)
AKI 1, adj A	0.83 (0.62-1.12)	ref	1.51 (1.21-1.86)
AKI 1, adj B	0.68 (0.51-0.92)	ref	1.13 (0.90-1.40)
	12 (3 events)	59 (17 events)	90 (51 events)
AKI 2	0.88 (0.26-3.01)	ref	2.48 (1.43-4.30)
AKI 2, adj A	0.83 (0.24-2.87)	ref	2.33 (1.33-4.07)
AKI 2, adj B	0.65 (0.19-2.23)	ref	1.55 (0.86-2.78)
	33 (17 events)	377 (223 events)	
AKI 3	0.85 (0.52-1.40)	ref	
AKI 3, adj A	1.67 (1.00-2.77)	ref	
AKI 3, adj B	1.52 (0.92-2.54)	ref	

Adj A = adjusted for age, gender and CCI. Adj B = adjusted for age, gender, CCI and type of intensive care unit.

Table S3b. Total mortality risk asin hazard ratios (95 % confidence intervals) when AKI is classified according to cystatin C, as compared to creatinine in the sub-cohort of patients with infections.

	Reclassified to lower AKI-stage by cystatin C	Not reclassified (same AKI-stage)	Reclassified to higher AKI-stage by cystatin C
AKI-staging by creatinine	HR (95 % CI)	HR (95 % CI)	HR (95 % CI)
		1384 (590 events)	565 (377 events)
AKI 0		ref	1.70 (1.48-1.94)
AKI 0, adj A		ref	1.49 (1.30-1.70)
AKI 0, adj B		ref	1.40 (1.22-1.60)
	92 (41 events)	129 (73 events)	315 (183 events)
AKI 1	0.70 (0.48-1.03)	ref	1.02 (0.78-1.34)
AKI 1, adj A	0.84 (0.57-1.24)	ref	1.11 (0.84-1.46)
AKI 1, adj B	0.76 (0.52-1.12)	ref	1.01 (0.77-1.33)
	9 (4 events)	18 (10 events)	74 (44 events)
AKI 2	0.58 (0.18-1.87)	ref	1.11 (0.56-2.21)
AKI 2, adj A	0.55 (0.17-1.77)	ref	1.19 (0.58-2.44)
AKI 2, adj B	0.46 (0.14-1.50)	ref	1.02 (0.50-2.10)
	33 (15 events)	261 (159 events)	
AKI 3	0.64 (0.38-1.09)	ref	
AKI 3, adj A	0.94 (0.55-1.62)	ref	
AKI 3, adj B	0.95 (0.55-1.64)	ref	

Adj A = adjusted for age, gender and CCI. Adj B = adjusted for age, gender, CCI and type of intensive care unit.

Table S4. Baseline characteristics of the 434 included in the validation cohort.

	Overall	AKI Stage based on creatinine 0	1	2	3	p
n	434	168	151	59	56	
Age (mean (SD))	56.10 (16.20)	55.79 (15.11)	54.69 (16.30)	56.61 (19.29)	60.30 (15.21)	0.169
Female gender, n (%)	290 (66.8)	105 (62.5)	111 (73.5)	35 (59.3)	39 (69.6)	0.104
Baseline Creatinine (mean (SD))	62.15 (43.49)	59.19 (20.02)	58.55 (31.12)	54.19 (23.95)	89.12 (97.75)	<0.001
Maximal Creatinine (mean (SD))	121.47 (121.46)	72.78 (19.59)	96.40 (43.65)	125.34 (57.83)	331.03 (229.24)	<0.001
Baseline Cystatin-C (mean (SD))	0.99 (0.63)	0.79 (0.32)	0.91 (0.48)	1.11 (0.68)	1.68 (1.01)	<0.001
Maximal Cystatin-C (mean (SD))	1.55 (1.06)	1.05 (0.36)	1.40 (0.75)	1.85 (1.01)	3.17 (1.49)	<0.001
Creatinine Stage (mean (SD))	1.01 (1.02)	0.00 (0.00)	1.00 (0.00)	2.00 (0.00)	3.00 (0.00)	<0.001
Cystatin-C Stage (mean (SD))	1.22 (1.17)	0.64 (0.86)	1.18 (1.09)	1.85 (1.13)	2.39 (1.02)	<0.001
1-year mortality, n (%)	54 (12.4)	15 (8.9)	20 (13.2)	6 (10.2)	13 (23.2)	0.041
Difference between Creatinine & Cystatin-C Stage (mean (SD))	0.21 (1.09)	0.64 (0.86)	0.18 (1.09)	-0.15 (1.13)	-0.61 (1.02)	<0.001
AKI stage based on creatinine, n (%)						<0.001
0	168 (38.7)	168 (100.0)	0 (0.0)	0 (0.0)	0 (0.0)	
1	151 (34.8)	0 (0.0)	151 (100.0)	0 (0.0)	0 (0.0)	
2	59 (13.6)	0 (0.0)	0 (0.0)	59 (100.0)	0 (0.0)	
3	56 (12.9)	0 (0.0)	0 (0.0)	0 (0.0)	56 (100.0)	
Myocardial infarction, n (%)	19 (4.4)	10 (6.0)	7 (4.6)	1 (1.7)	1 (1.8)	0.402
Congestive Heart Failure, n (%)	16 (3.7)	3 (1.8)	10 (6.6)	2 (3.4)	1 (1.8)	0.114
Peripheral vascular disease, n (%)	15 (3.5)	4 (2.4)	4 (2.6)	0 (0.0)	7 (12.5)	0.001
Cerebrovascular disease, n (%)	170 (39.2)	90 (53.6)	58 (38.4)	16 (27.1)	6 (10.7)	<0.001
Dementia, n (%)	0 (0.0)	0 (0.0)	0 (0.0)	0 (0.0)	0 (0.0)	NA
Chronic Obstructive Pulmonary Disease, n (%)	17 (3.9)	7 (4.2)	5 (3.3)	3 (5.1)	2 (3.6)	0.939
Other chronic pulmonary disease, n (%)	22 (5.1)	5 (3.0)	9 (6.0)	5 (8.5)	3 (5.4)	0.36
Rheumatic disease, n (%)	3 (0.7)	1 (0.6)	0 (0.0)	2 (3.4)	0 (0.0)	0.052
Peptic ulcer, n (%)	4 (0.9)	1 (0.6)	0 (0.0)	2 (3.4)	1 (1.8)	0.112
Mild liver disease, n (%)	6 (1.4)	1 (0.6)	3 (2.0)	0 (0.0)	2 (3.6)	0.265
Diabetes mellitus, n (%)	1 (0.2)	0 (0.0)	1 (0.7)	0 (0.0)	0 (0.0)	0.598
Hemiplegia, n (%)	28 (6.5)	7 (4.2)	11 (7.3)	5 (8.5)	5 (8.9)	0.458
Severe kidney disease, n (%)	15 (3.5)	1 (0.6)	4 (2.6)	1 (1.7)	9 (16.1)	<0.001
Diabetes mellitus with end-organ disease, n (%)	3 (0.7)	0 (0.0)	1 (0.7)	1 (1.7)	1 (1.8)	0.389
Malignancy, n (%)	39 (9.0)	10 (6.0)	17 (11.3)	4 (6.8)	8 (14.3)	0.163
Severe liver disease, n (%)	0 (0.0)	0 (0.0)	0 (0.0)	0 (0.0)	0 (0.0)	NA
Metastatic cancer, n (%)	8 (1.8)	3 (1.8)	3 (2.0)	0 (0.0)	2 (3.6)	0.562
AIDS, n (%)	0 (0.0)	0 (0.0)	0 (0.0)	0 (0.0)	0 (0.0)	NA
Max Charlson Comorbidity Index at baseline (mean (SD))	0.00 (0.00)	0.00 (0.00)	0.00 (0.00)	0.00 (0.00)	0.00 (0.00)	NA

Table S5. Cox proportional hazards model for mortality during follow-up with adjustment for ICU type as a random effect.

Covariate	HR 95% CI
AKI Stage	1.28 (1.23–1.33)
Reclassification	1.343 (1.30–1.39)
Female gender	1.02 (0.96–1.09)
Age	1.88 (1.81–1.95)
Type of ICU (frailty term)	Variance = 1.098

AKI: Acute kidney injury, HR: Hazard ratio, CI: Confidence interval

Table S6. Cox proportional hazards model for mortality during follow-up when AKI is classified according to cystatin C in the validation cohort. The first creatinine after admission was included in the model.

Variable	HR (95% CI)
AKI Stage	1.52(1.16-1.99)**
Reclassification to a higher AKI stage	1.43 (1.12-1.81)**
Age	1.06 (0.79-1.42)
Female gender	0.90 (0.50-1.62)
First plasma creatinine	0.99 (0.98-0.99)*

AKI: Acute kidney injury, HR: Hazard ratio, CI: Confidence interval
